# Resolving Bivalirudin Interference on Fibrinogen Testing by the Use of Activated Carbon

**DOI:** 10.1002/jcla.70184

**Published:** 2026-02-18

**Authors:** Jia Du, Haiyan Liu, Xiaohui Liu, Shuzheng Cao, Zhenlu Zhang, Litao Zhang

**Affiliations:** ^1^ Clinical Laboratory Wuhan Asia General Hospital Affiliate to Wuhan University of Science and Technology Wuhan China; ^2^ Center of Laboratory Medicine Wuhan Asia Heart Hospital Affiliate to Wuhan University of Science and Technology Wuhan China

**Keywords:** activated carbon, Clauss method, direct thrombin inhibitor, heparin induced thrombocytopenia, thrombin

## Abstract

**Background:**

Direct thrombin inhibitors may lead to falsely low fibrinogen results. The interference of bivalirudin on fibrinogen assays has not been thoroughly evaluated. This study investigates the interference of bivalirudin on fibrinogen detection and the potential use of activated carbon to address this issue.

**Methods:**

Normal pooled plasma from 20 healthy subjects was spiked with increasing bivalirudin concentrations (0–6.4 μg/mL). Interference of bivalirudin on fibrinogen testing was evaluated by comparing three Clauss‐method assays (HemosIL Fibrinogen‐C XL; STA‐Fibrinogen; Dade Thrombin) to an immunoassay (N Antiserum to Human Fibrinogen reagent, NAHF). Activated partial thromboplastin time (APTT) and fibrinogen results were analyzed before and after the addition of activated carbon (AC) to evaluate the absorption of bivalirudin by AC.

**Results:**

The NAHF immunoassay remained unaffected by bivalirudin (*p* = 0.27). All three Clauss‐methods assays showed a significant downward trend with the increase of bivalirudin concentration (STA‐Fibrinogen: *p* = 0.012, Dade Thrombin: *p* < 0.001, Fibrinogen‐C XL: *p* < 0.001). When compared to their respective baselines, STA‐Fibrinogen exhibited a reduction of 16%, Dade Thrombin showed a reduction of 40%, and Fibrinogen‐C XL displayed a decrease of up to 89%. Neither STA‐Fibrinogen nor Dade Thrombin displayed a significant downward trend when the APTT ratio did not exceed 2.5. After the addition of AC, SynthASil APTT and HemosIL Fibrinogen‐C XL levels of all samples containing varying concentrations of bivalirudin returned to near baseline.

**Conclusion:**

The influences of bivalirudin on fibrinogen assays vary significantly. Activated carbon can effectively remove bivalirudin from plasma, thereby eliminating its interference on fibrinogen testing.

## Graphical abstract

The influences of bivalirudin on fibrinogen assays differ considerably. Activated carbon can effectively remove bivalirudin from plasma, thereby eliminating its interference in fibrinogen detection.

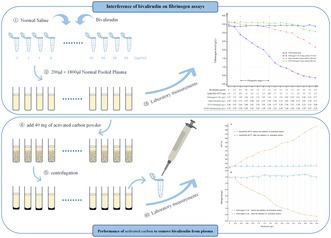

## 1 Introduction

Bivalirudin, a synthetic polypeptide with a molecular weight of 2180 Da, exerts direct inhibition on thrombin by simultaneously blocking its active site and fibrinogen binding site. As a parenteral direct thrombin inhibitor (DTI), it has gained increasing usage in patients undergoing percutaneous coronary intervention procedures [1, 2], those with heparin‐induced thrombocytopenia (HIT) [3], or requiring extracorporeal life support (ECLS) [4].

Monitoring anticoagulation levels—typically by Activated Clotting Time (ACT) or Activated Partial Thromboplastin Time (APTT) ratio—and assessing the coagulation status are essential during bivalirudin infusion. Fibrinogen level is one of the most commonly assessed coagulation tests, predominantly detected using Clauss‐method based assays in current laboratories [5, 6]. Previous studies have reported that DTIs can lead to false low fibrinogen results due to their inhibition of thrombin used in fibrinogen assay [7, 8, 9]. Our previous study demonstrated considerable variation in argatroban interference on fibrinogen assays due to differing concentrations of thrombin employed in commercial fibrinogen reagents [7]. However, as bivalirudin is also a DTI and shares similar mechanisms with argatroban, the interference of bivalirudin on fibrinogen testing has not been systematically evaluated yet.

Meanwhile, activated carbon (AC) is widely used to treat intoxications for its absorbability [10]. Numerous studies have demonstrated its efficacy in removing direct oral anticoagulants (such as dabigatran and rivaroxaban) from plasma [11]. However, it remains unknown whether AC can effectively remove bivalirudin from plasma to resolve the interference of bivalirudin on coagulation assays. In this study, we conducted an in vitro investigation aiming to test our hypothesis and explore potential solutions.

## 2 Methods

### 2.1 Samples Preparation

Whole blood samples from 20 healthy subjects were collected into coagulation tubes containing 3.2% sodium citrate (blood/citrate, 9:1 v/v; BD Vacutainer, Plymouth, UK). The samples underwent immediate centrifugation at 2500 *g* for 10 min to isolate the supernatant plasma. The supernatant plasma was thoroughly mixed to create the normal pooled plasma (NPP). Bivalirudin (Qiwen, Qilu Pharmaceutical, China) was diluted with normal saline (NS) to create a standard series ranging from 0 μg/mL (NS) to 64 μg/mL. To achieve final bivalirudin plasma concentrations ranging from 0 to 6.4 μg/mL, 1800 μL of NPP samples were spiked with 200 μL of the standard series. Prior ethical committee approval and informed consent from all subjects were obtained before commencing the study.

### 2.2 Laboratory Measurements

Firstly, all samples were analyzed using four fibrinogen assays within 2 h after preparation. The fibrinogen assays used included HemosIL Fibrinogen‐C XL reagent (Clauss method) with a thrombin concentration of 35 NIH U/mL on ACL‐TOP analyzer (both from Werfen, USA); STA‐Fibrinogen reagent (Clauss method) with a thrombin concentration of 80 NIH U/mL on STA‐R Evolution analyzer (both from Diagnostica Stago, France); Dade Thrombin reagent (Clauss method, Siemens Healthcare, Germany) with a thrombin concentration of 100 NIH U/ml on CS‐5100 analyzer (Sysmex Corporation, Japan); and N Antiserum to Human Fibrinogen reagent (NAHF Immunoassay) on BN2 nephelometer analyzer (both from Siemens Healthcare, Germany).

Subsequently, 40 mg of AC powder (Beijing Solarbio Science & Technology Co. Ltd., China) was added to each sample, followed by gentle mixing for 5 min. After centrifugation at 2500 *g* for 10 min, the supernatant was transferred to new tubes. Finally, the removal efficiency of AC on bivalirudin was assessed by re‐measuring fibrinogen levels in the residual plasma. The HemosIL Fibrinogen‐C XL assay on the ACL‐TOP analyzer was selected as the primary indicator for this assessment due to its low thrombin concentration (35 NIH U/mL), which makes it highly sensitive to DTI interference and thus provides the most stringent test for evaluating the efficacy of AC removal.

Simultaneously, APTT was measured using SynthASil APTT reagent on ACL‐TOP analyzer (both from Werfen, USA). All tests were performed according to the original manufacturer's instructions. The local reference range for APTT is between 25.6 and 36.5 s. The baseline APTT value for NPP was 29.4 s.

### 2.3 Data Analysis and Statistics

Fibrinogen results and APTT ratios (calculated using 29.4 s as baseline) were expressed as arithmetical means. The interference of bivalirudin on immunoassay was evaluated by *single sample t*‐test. Trends of results between immunoassay and other fibrinogen assays were compared, respectively, by *Cochran‐Armitage* test. A *p* < 0.05 was considered to be statistically significant. The data were analyzed by MedCalc (MedCalc Statistical Software version 16.2.1, Ostend, Belgium).

## 3 Results

### 3.1 Interference of Bivalirudin on Fibrinogen Assays

**FIGURE 1:**
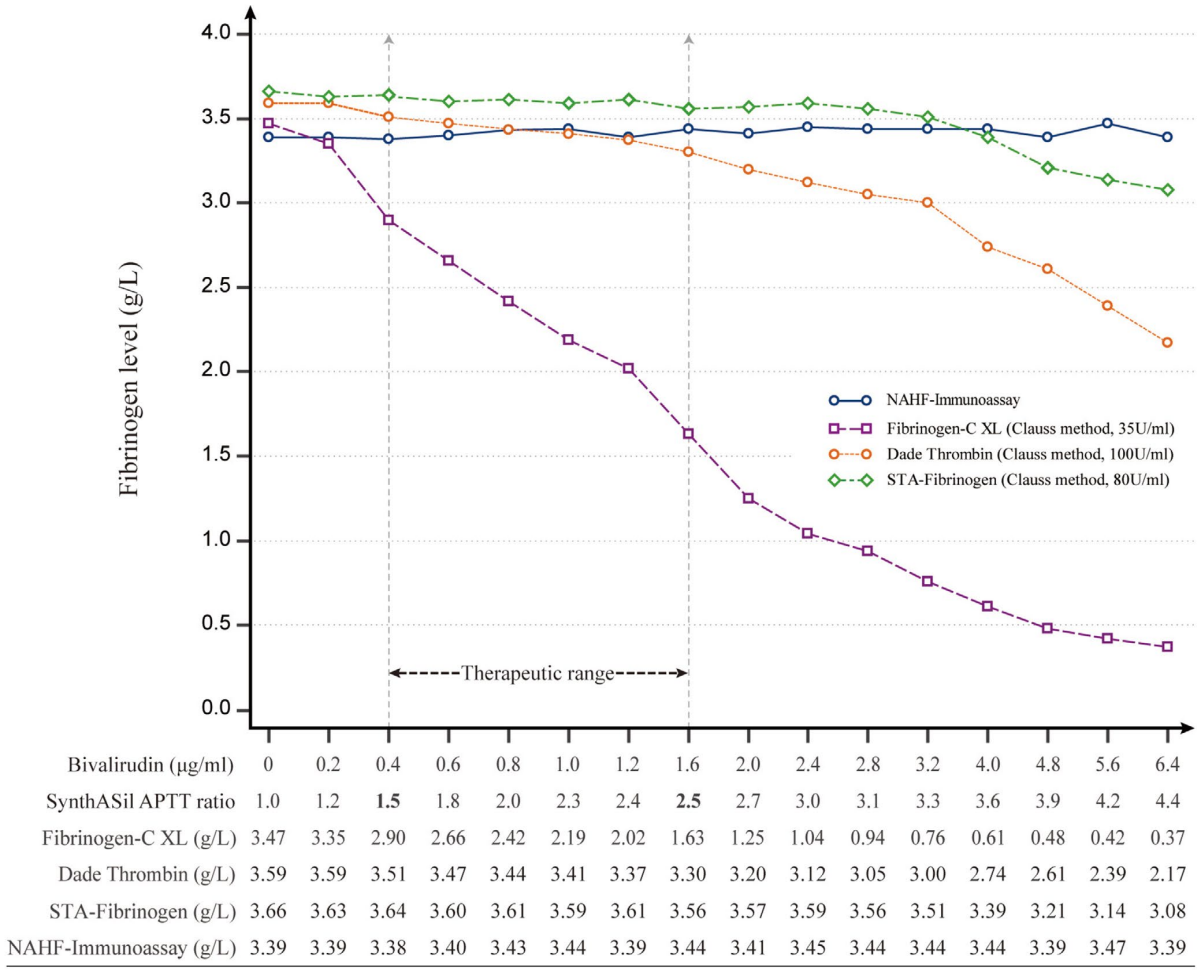
Trends of results from four fibrinogen assays. As the concentration of bivalirudin increased, the APTT ratio increased accordingly. The fibrinogen results in each column were aligned with the corresponding APTT ratio listed above. The fibrinogen baseline values obtained from bivalirudin‐free samples (APTT ratio: 1.0) were listed in the first column. APTT, Activated partial thromboplastin time; NAHF, N Antiserum to Human Fibrinogen reagent.

The results and trends of fibrinogen in relation to different concentrations of bivalirudin are illustrated in Figure 1. As the concentration of bivalirudin increased, the APTT ratio also increased (APTT ratio 1.5 at 0.4 μg/mL bivalirudin; APTT ratio 4.4 at 6.4 μg/mL bivalirudin, Figure 1). NAHF remained unaffected by bivalirudin (*p* = 0.27). Compared to the immunoassay, when the APTT ratio reached 4.4 (at a concentration of 6.4 μg/mL bivalirudin), all three Clauss‐method assays exhibited significantly lower results (STA‐Fibrinogen: *p* = 0.012, Dade Thrombin: *p* < 0.001, Fibrinogen‐C XL: *p* < 0.001, Figure 1). Specifically, when compared to their baseline fibrinogen values, STA‐Fibrinogen exhibited a reduction of 16%, Dade Thrombin showed a reduction of 40%, and Fibrinogen‐C XL displayed an impressive decrease of up to 89%. Notably, both STA‐Fibrinogen and Dade Thrombin did not exhibit significant downward trend when the APTT ratio did not exceed 2.5 (*p* = 0.66, 0.22, respectively). In contrast, the HemosIL Fibrinoen‐C XL reagent exhibited a substantial decline from the beginning (APTT ratio: l.2 at 0.2 μg/mL bivalirudin, Figure 1).

### 3.2 Performance of AC to Remove Bivalirudin From Plasma

**FIGURE 2:**
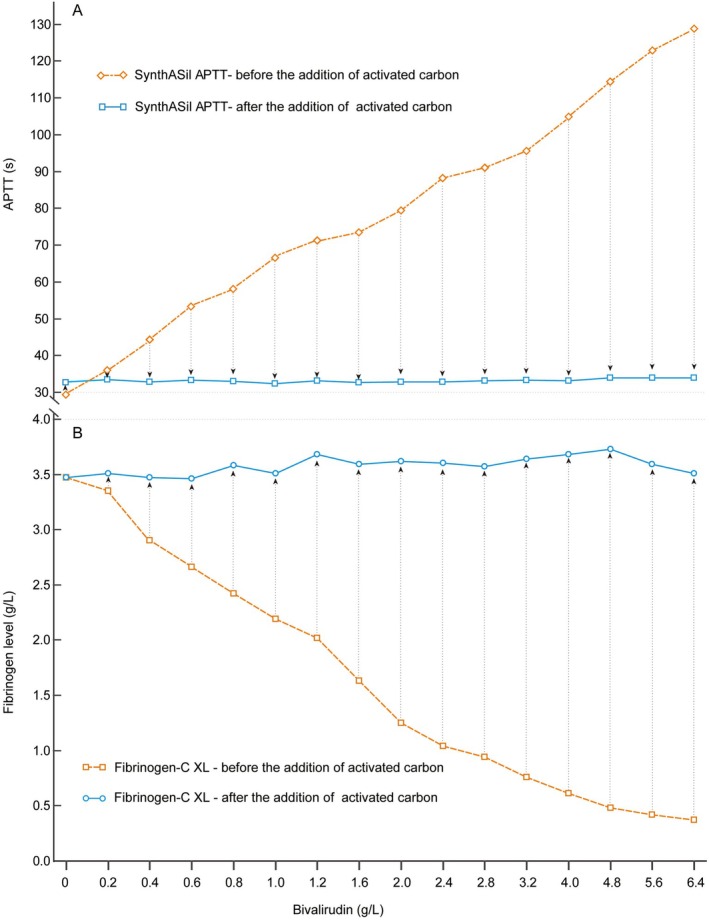
SynthASil APTT (seconds) and HemosIL Fibrinogen‐C XL (g/L) results before and after the addition of activated carbon. After the addition of activated carbon, all SynthASil APTT and HemosIL Fibrinogen‐C XL results returned nearly to their baseline levels. APTT, activated partial thromboplastin time.

Figure 2 illustrates the removal of bivalirudin by AC. After the addition of AC, SynthASil APTT results of all samples with a series of bivalirudin decreased to within the normal range (33.2 ± 0.47 s, Figure 2A). Even in the sample with the highest concentration of bivalirudin, SynthASil APTT dropped from 130 s (APTT ratio: 4.4) to 33.9 s (APTT ratio: 1.0). Simultaneously, fibrinogen levels measured using Fibrinogen‐C XL reagents, which were most affected by the drug, also returned to near baseline (Figure 2B), including in the sample with the highest concentration of bivalirudin (from 0.37 to 3.51 g/L). The above findings demonstrate that AC can effectively remove bivalirudin from plasma.

## 4 Discussion

The primary finding of the current study reveals that bivalirudin has the potential to induce false low fibrinogen results, which may lead to misdiagnoses. However, the effects of bivalirudin on fibrinogen assays exhibit significant variability. Moreover, AC can effectively eliminate the interference of bivalirudin with fibrinogen detection by removing it from plasma, which might be useful for fibrinogen detection or reversal during the management of patients undergoing bivalirudin therapy.

The mechanism underlying the generation of false low fibrinogen results by DTIs involves their inhibition of thrombin within the fibrinogen reagent, thereby prolonging clot formation. Previous reports found that all DTIs (argatroban, bivalirudin, dabigatran, etc.) can cause this interference [7, 12, 13]. Molinaro et al. [8] reported a significant influence of bivalirudin on fibrinogen levels, with even Dade‐Behring Thrombin reagent yielding results below detection limits (< 0.6 g/L) at a concentration of 10 μg/mL of bivalirudin, which aligns with our current study findings. Nevertheless, heterogeneity exists among DTIs regarding their influence on fibrinogen assays and can be attributed to two factors:

Firstly, during Clauss fibrinogen assessments, a high concentration of thrombin is added to diluted plasma [6]. Therefore, the effects of bivalirudin on Clauss fibrinogen assays depend on both the concentrations of bivalirudin and thrombin within the reaction system. There is considerable variation in the thrombin concentrations utilized in commercial fibrinogen assays, ranging from 35 to 200 U/mL [5, 7], which may result in variable effects of DTIs on fibrinogen measurements. In the current study, bivalirudin showed the strongest interference on Fibrinogen‐C XL reagent with 35 U/mL of thrombin (Figure 1), compared to the other two reagents using higher concentrations of thrombin, even though the other two reagents did not exhibit a significant downward trend under 2.5 of APTT ratio, which is the upper limit of the therapeutic range for patients with HIT (APTT ratio: 1.5–2.5) [3]. This suggested that institutions should investigate reagents in use in on‐site laboratories as the interference is likely dependent on the thrombin concentration of the reagent.

Interestingly, bivalirudin had a smaller effect on STA‐Fibrinogen compared to the Dade Thrombin reagent, even though the thrombin concentration in STA‐Fibrinogen (80 U/mL) was lower than in Dade Thrombin (100 U/mL). Both Molinaro et al. [8] and our observations [7] noted that DTIs, including argatroban, showed this pattern. Molinaro et al. attributed this difference to the distinct clot detection methods employed by the two systems [8]. Specifically, the Dade‐Behring system measures fibrinogen levels through changes in photo‐optical turbidity, whereas the STA‐R system detects increased viscosity from clotting by monitoring the pendulum motion of a steel ball (see Table 1) [5]. In fact, this effect is likely due to differences in the dilution ratios of the test plasma [6]. As detailed in Table 1, the dilution ratios used by the original instruments for fibrinogen measurement are 1:10 for Dade Thrombin and 1:20 for STA‐Fibrinogen.

**TABLE 1:** The parameters of four fibrinogen assays.

Instrument	Fibrinogen reagent	Testing principle	Detection method	Dilution ratio	Thrombin concentration (U/mL)	Thrombin source
ACL‐TOP	Fibrinogen‐C XL	Clauss method	Photo‐optical	1:10	35	Bovine
STA‐R Evolution	STA‐Fibrinogen	Clauss method	Steel ball–mechanical	1:20	80	Human
CS‐5100	Dade Thrombin	Clauss method	Photo‐optical	1:10	100	Bovine
BN2	N Antiserum to Human Fibrinogen	Immunoassay	Immuno‐turbidimetry	1:20	/	/

Secondly, the degree of effects among DTIs differs greatly. Molinaro et al. [8] demonstrated that fibrinogen measurements are affected more by argatroban than by bivalirudin for the Clauss method, which is consistent with our previous and current studies. The mechanism of this phenomenon is still unclear. It might be due to the fact that the molecular weight of argatroban (527 Da) is much smaller than that of bivalirudin (2180 Da), resulting in more inhibition of exogenously added thrombin for the fibrinogen assay when there is a molar excess of argatroban [5, 7]. Additionally, STA‐Fibrinogen uses human thrombin while Fibrinogen‐C XL and Dade thrombin use bovine thrombin. The affinity between DTIs and thrombin from different origins may vary [5]. However, these conjectures need further confirmation.

Currently, there are no specific antagonists available for bivalirudin [14], either in vivo or in vitro. Our study is the first to demonstrate that AC could efficiently remove bivalirudin from plasma. This finding suggests that AC may have potential clinical applications: (1) Reversal of bivalirudin in vivo. In certain situations, reversal of bivalirudin is necessary. Previous report has shown that haemofiltration and plasmapheresis can only remove 65%–69% [15]. Combining AC with these treatments may potentially enhance treatment clearance efficacy. Interestingly, a similar adsorbing effect by AC was observed for argatroban (Du J, Liu X, Zhang L, unpublished data). (2) In vitro experiments indicate three methods to obtain accurate results when detecting fibrinogen in the presence of DTIs: (a) utilizing immunoassay; (b) employing AC to eliminate DTIs prior to detection; and (c) if the APTT ratio is lower than 2.5, using reagents with very high concentrations of thrombin (equivalent concentration of 100 U/mL or more).

This study has two limitations. Firstly, this was an in vitro study that recruited healthy subjects, which may differ from the in vivo response in patients. Secondly, we utilized APTT ratio to monitor bivalirudin. Although APTT is recommended by guidelines for bivalirudin monitoring, using calibrated quantitative method would yield more direct supports [16].

## 5 Conclusion

The influences of bivalirudin on fibrinogen assays differ considerably. Clinicians should be aware of these influences to avoid misdiagnoses. Activated carbon can effectively remove bivalirudin from plasma, thereby eliminating its interference in fibrinogen detection. This approach may prove valuable for assessing fibrinogen levels or drug reversing in patients undergoing bivalirudin therapy.

## Author Contributions

Litao Zhang designed the study and wrote the initial draft of the manuscript. Jia Du performed the research, collected, and analyzed the data. Haiyan Liu, Xiaohui Liu, and Shuzheng Cao performed the research and collected the data. Zhenlu Zhang reviewed and edited the manuscript. Litao Zhang obtained the funding.

## Funding

This work was supported by Health and Family Planning Commission of Wuhan Municipality (WX20C23).

## Conflicts of Interest

The authors declare no conflicts of interest.

## Data Availability

The data that support the findings of this study are available from the corresponding author upon reasonable request due to privacy or ethical restrictions.

## References

[1] Y. Li , Z. Liang , L. Qin , et al., “Bivalirudin Plus a High‐Dose Infusion Versus Heparin Monotherapy in Patients With ST‐Segment Elevation Myocardial Infarction Undergoing Primary Percutaneous Coronary Intervention: A Randomised Trial,” Lancet 10366 (2022): 1847–1857. 10.1016/S0140-6736(22)01999-7 36351459

[2] D. Erlinge , E. Omerovic , O. Frobert , et al., “Bivalirudin Versus Heparin Monotherapy in Myocardial Infarction,” New England Journal of Medicine 12 (2017): 1132–1142. 10.1056/NEJMoa1706443 28844201

[3] A. Cuker , G. M. Arepally , B. H. Chong , et al., “American Society of Hematology 2018 Guidelines for Management of Venous Thromboembolism: Heparin‐Induced Thrombocytopenia,” Blood Advances 22 (2018): 3360–3392. 10.1182/bloodadvances.2018024489 PMC6258919 30482768

[4] J. Helms , C. Frere , T. Thiele , et al., “Anticoagulation in Adult Patients Supported With Extracorporeal Membrane Oxygenation: Guidance From the Scientific and Standardization Committees on Perioperative and Critical Care Haemostasis and Thrombosis of the International Society on Thrombosis and Haemostasis,” Journal of Thrombosis and Haemostasis 2 (2023): 373–396. 10.1016/j.jtha.2022.11.014 36700496

[5] L. J. Stang and L. G. Mitchell , “Fibrinogen,” Methods in Molecular Biology 992 (2013): 181–192. 23546714 10.1007/978-1-62703-339-8_14

[6] J. Yan , L. Liao , D. Deng , et al., “Guideline for Diagnosis and Management of Congenital Dysfibrinogenemia,” Clinica Chimica Acta 561 (2024): 119680. 10.1016/j.cca.2024.119680 38642629

[7] L. Zhang , J. Yang , X. Zheng , Q. Fan , and Z. Zhang , “Influences of Argatroban on Five Fibrinogen Assays,” International Journal of Laboratory Hematology 6 (2017): 641–644. 10.1111/ijlh.12719 28766891

[8] R. J. Molinaro , F. Szlam , J. H. Levy , C. R. Fantz , and K. A. Tanaka , “Low Plasma Fibrinogen Levels With the Clauss Method During Anticoagulation With Bivalirudin,” Anesthesiology 1 (2008): 160–161. 10.1097/ALN.0b013e31817885b7 18580197

[9] I. Mackie , A. Casini , M. Pieters , et al., “International Council for Standardisation in Haematology Recommendations on Fibrinogen Assays, Thrombin Clotting Time and Related Tests in the Investigation of Bleeding Disorders,” International Journal of Laboratory Hematology 1 (2024): 20–32. 10.1111/ijlh.14201 37984807

[10] T. Zellner , D. Prasa , E. Farber , et al., “The Use of Activated Charcoal to Treat Intoxications,” Deutsches Ärzteblatt International 18 (2019): 311–317. 10.3238/arztebl.2019.0311 PMC6620762 31219028

[11] G. Frans , P. Meeus , and E. Bailleul , “Resolving DOAC Interference on aPTT, PT, and Lupus Anticoagulant Testing by the Use of Activated Carbon,” Journal of Thrombosis and Haemostasis 8 (2019): 1354–1362. 10.1111/jth.14488 31102433

[12] I. Jennings , W. Lester , E. Gray , et al., “Effect of Direct Thrombin Inhibitors on Laboratory Measurement of Fibrinogen: Potential for Errors in Clinical Decision‐Making,” International Journal of Laboratory Hematology 4 (2023): 599–602. 10.1111/ijlh.14040 36793181

[13] T. L. Lindahl , F. Baghaei , I. F. Blixter , et al., “Effects of the Oral, Direct Thrombin Inhibitor Dabigatran on Five Common Coagulation Assays,” Thrombosis and Haemostasis 2 (2011): 371–378. 10.1160/TH10-06-0342 21103660

[14] E. M. Van Cott , A. J. Roberts , and W. E. Dager , “Laboratory Monitoring of Parenteral Direct Thrombin Inhibitors,” Seminars in Thrombosis and Hemostasis 3 (2017): 270–276. 10.1055/s-0036-1597297 28052306

[15] A. Koster , D. Chew , M. Grundel , et al., “An Assessment of Different Filter Systems for Extracorporeal Elimination of Bivalirudin: An In Vitro Study,” Anesthesia and Analgesia 5 (2003): 1316–1319. 10.1213/01.ANE.0000057605.61063.55 12707125

[16] J. T. Beyer , S. E. Lind , S. Fisher , et al., “Evaluation of Intravenous Direct Thrombin Inhibitor Monitoring Tests: Correlation With Plasma Concentrations and Clinical Outcomes in Hospitalized Patients,” Journal of Thrombosis and Thrombolysis 2 (2020): 259–267. 10.1007/s11239-019-01961-3 31559512

